# Mirna interference in human pulmonary alveolar epithelial cells (HPAEPIC) undergoing cyclic stretch and in *ex vivo* ventilated and perfused rat lungs

**DOI:** 10.1186/2197-425X-3-S1-A566

**Published:** 2015-10-01

**Authors:** A Ferruelo, B Olaiz, R Herrero, E Lopez, A Esteban, JÁ Lorente

**Affiliations:** Hospital Universitario de Getafe, Getafe, Spain; Ciber de Enfermedades Respiratorias, Madrid, Spain; Universidad Europea, Madrid, Spain

## Introduction

We have previously demonstrated that the expression of miRNA 27a-5p is associated with DAD in an experimental model of ventilator-induced lung injury and in patients with ARDS.

## Objectives

To modulate miRNA 27a-5p expression in HPAEPiC undergoing stretch and in *ex vivo* ventilated perfused lungs.

## Methods

HPAEPiCs were transfected overnight with miRNA 27a-5p inhibitor (10 nM, 20 nM and 50 nM) or mimic (20 nM, 40 nM) or the negative control inhibitor (Exiqon) using HiPerfect transfection agent and underwent cyclic stretch for 6 h (15% linear elongation, 0.2 Hz). Cells were then cultured for up to 48 h for miRNA isolation (miRNeasy Mini Kit, Qiagen) and for 72 h for protein isolation (T-Per, Pierce). All assays were performed in triplicate.

Adult male Sprague-Dawley rats (weight 325-375 g) were anesthetized and sacrificed by exsanguination, and the heart and lung were extracted en bloc and mounted in a ventilation chamber for perfusion (Krebs solution) and ex vivo ventilation for 2.5 h (VT = 6 mL/kg, PEEP = 5 cm H2O (Harvard Apparatus, MA). miRNA 27a-5p inhibitor (0.25 and 0.50 mg/kg lung weight), its corresponding controls, as well as miRNA 27a-5p mimic (0.10 mg/kg) were administered intratracheally 30 min before mechanical ventilation (n = 5 for each of the 5 groups).

Expression of miRNA 27a-5p was quantified by RT-qPCR (miScript II RT and QuantiTect SYBR Green PCR, Qiagen) in a 7500 Fast Real-Time PCR (Life Technologies) and data were analyze with the ΔΔCT method. Epidermal growth factor receptor (EGFR) protein concentration, a miRNA 27a-5p target, was measured in cell and tissue lysates for Elisa.

Values were compared by the Kruskal-Wallis method. The study was conducted with the approval of the local IRB. Values are fold change as compared to the negative control, or median (IQR). p < 0.05 was considered statistically significant.

## Results

Treatment with the miRNA 27a-5p inhibitor dose dependently decreased miRNA 27a-5p expression vs. negative control (10 nM: 1.05; 20 nM: 0.80; 50 nm: 0.62); and increased EGFR concentration vs negative control (10 nM: 1.20; 20 nM: 1.36; 50 nM: 1.45). Treatment with the miRNA 27a-5p mimic increased miRNA 27a-5p expression (20 nM: 150; 40 nM: 200) and decreased EGFR concentration (20 nM: 0.79; 40 nM: 0.78).

In lung tissue, miRNA 27a-5p inhibitor down-regulated miRNA 27a-5p expression (46 × 10-4 (42 × 10^-4^-54 × 10^-4^) vs. 15 × 10^-4^ (10 × 10^-4^-54 × 10^-4^) and 76 × 10^-4^ (57 × 10^-4^-93 × 10^-4^) vs. 11 × 10^-4^ (8 × 10^-4^-20 × 10^-4^)), for the 0.25 mg/kg and 0.50 mg/kg doses, respectively. EGFR concentration increased with the 0.50 mg/kg dose (0.17 (0.41-0.47) vs. 0.42 (0.41-0.47) pg/ug protein).

miRNA 27a-5p mimic up-regulated miRNA 27a-5p expression (3324 × 10^-4^ (2023 × 10^-4^-4825 × 10^-4^) vs 46 × 10^-4^ (42 × 10^-4^-54 × 10^-4^)) and reduced EGFR concentration (0.34 (0.22-0.43) vs. 0.11 (0.08-0.20).

## Conclusions

It is possible to modulate in vitro and ex vivo the expression of miRNA 27a-5p, a miRNA associated with DAD.

## Funding

Fis PI12/2898 and FEDER Funds

